# Uncovering distinct clinical phenotypes in disseminated intravascular coagulation through machine learning-enabled cluster analysis

**DOI:** 10.3389/fmolb.2026.1699476

**Published:** 2026-02-25

**Authors:** Qingbo Zeng, Junjie Zeng, Qingwei Lin, Lincui Zhong, Longping He, Jingchun Song

**Affiliations:** 1 Intensive Care Unit, The 908th Hospital of Chinese PLA Logistic Support Force, Nanchang, China; 2 Intensive Care Unit, Nanchang Hongdu Hospital of Traditional Chinese Medicine, Nanchang, China; 3 Nanchang Key Laboratory of Thrombosis and Hemostasis, Nanchang, China

**Keywords:** cluster analysis, disseminated intravascular coagulation, machine learning, phenotypes, stratification

## Abstract

**Background:**

Disseminated intravascular coagulation (DIC) is a critical condition encountered in the intensive care unit (ICU), characterized by multiple etiologies and variable outcomes. Distinguishing between DIC phenotypes poses a significant challenge. This study aims to apply unsupervised machine learning (ML) algorithms to stratify DIC patients, thereby enabling more personalized treatment approaches.

**Methods:**

We conducted a retrospective analysis of patients diagnosed with DIC upon admission to the ICU at a comprehensive teaching tertiary hospital in China, spanning from May 2015 to November 2022. We applied an unsupervised machine learning approach for consensus clustering using the R package Consensus Cluster Plus to identify clinical phenotypes in 134 patients with DIC. The analysis incorporated the key variables: Thrombin-Antithrombin Complex (TAT), Plasmin-α_2_-Plasmin Inhibitor Complex (PIC), tissue plasminogen activator-inhibitor complex (tPAIC), and thrombomodulin (TM). The elbow method, cumulative distribution function (CDF) plot, and consensus matrix were employed to ascertain the optimal number of clusters. Logistic regression (LR) analysis was used to investigate the association between the identified phenotypes and clinical endpoints.

**Results:**

The consensus cluster analysis delineated two distinct subtypes: a mild coagulation dysfunction subtype (n = 79) and a severe coagulation dysfunction subtype (n = 55). Notable differences were observed in both variables included in the analysis (e.g., thrombin-antithrombin complex [TAT], *P < 0.05*) and those not utilized for model training (e.g., heart rate [HR] *P < 0.05* and systolic blood pressure [SBP] *P < 0.05*). Logistic regression revealed that the severe coagulation dysfunction subtype was significantly associated with increased odds of 7-day (OR 4.71; 95% CI 2.23–9.98; *P < 0.001*), 28-day (OR 2.29; 95% CI 1.11–4.72; *P = 0.024*).

**Conclusion:**

The study identified two clusters with distinct laboratory profiles and mortality risk.

## 1 Introduction

Disseminated intravascular coagulation (DIC) is a critical complication in the critically ill, often precipitated by severe infections, trauma, malignancies, liver diseases, obstetric emergencies, or as a consequence of progressive severe coagulopathy (Levi and Sivapalaratnam, 2018; Papageorgiou et al., 2018). The pathophysiology of DIC is intricate, with pivotal factors such as impaired fibrinolysis, tissue factor-mediated intravascular coagulation, and compromised anticoagulant mechanisms (Wada et al., 2014). Despite recent therapeutic advancements, DIC continues to carry a distressingly high mortality rate, which exhibits considerable variation, with 30-day mortality rates ranging from 20% to 45% (Adelborg et al., 2021; Bakhtiari et al., 2004; Gando et al., 2008). This variability suggests that DIC patients constitute a heterogeneous group, with distinct phenotypes that differ markedly in clinical presentation and outcomes, necessitating separate consideration in clinical practice and research. Current DIC classification schemes predominantly rely on expert consensus and opinion (Wada and Gando, 2024; Matsuda and Kurokawa, 2020; Ohbe et al., 2020). Consequently, there is an imperative for a more nuanced classification of the DIC spectrum to enhance the crucial selection of appropriate management strategies, particularly in identifying optimal candidates for advanced therapeutic interventions.

As electronic medical records (EMR) and artificial intelligence progress, machine learning (ML) algorithms are increasingly utilized in personalized medicine to enhance clinical decision-making (MacEache et al., 2021; Sohail and Arif, 2020; Fröhlich et al., 2018). One such technique, consensus clustering, an unsupervised ML method, identifies patterns of similarity and dissimilarity across multiple variables, thereby grouping them into distinct phenotypes (Brière et al., 2021). This method generates multiple clustering outcomes over several iterations and synthesizes them into a final result, providing a visual representation that enhances the understanding and interpretability of the algorithm. Studies have demonstrated its efficacy in discerning clinically distinct disease phenotypes, including those in cardiovascular diseases (Min et al., 2024).

Given the heterogeneity observed in patients with DIC upon admission (Okajima et al., 2000), our study aimed to identify clinically meaningful phenotypes among DIC patients using an unsupervised ML approach and to evaluate the mortality risks associated with these distinct clusters.

## 2 Methods

### 2.1 Study design and participant cohort

**FIGURE 1:**
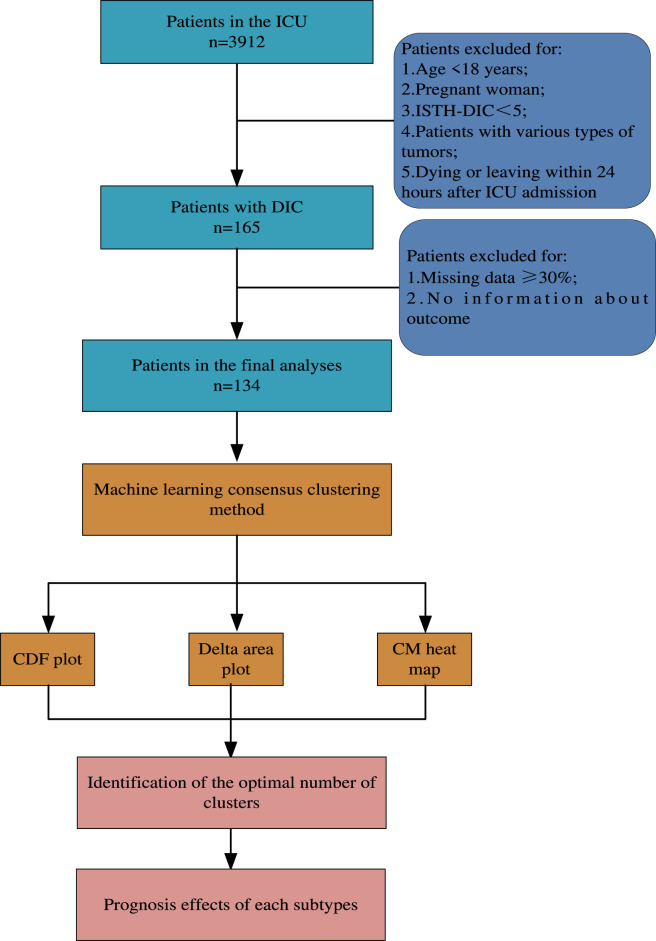
Flow chart of patients with DIC. Abbreviations: CDF plot, Cumulative Distribution Function Plot; CM heat map, Confusion Matrix Heat Map.

This investigation leverages data procured from the 908th Hospital of the PLA Logistical Support Force, encompassing a period from May 2015 to November 2022. The study sample comprised 3,912 individuals. Participant exclusion criteria were as follows: (1) age below 18 years; (2) pregnant status; (3) the International Society of Thrombosis and Hemostasis (ISTH)-DIC score below the threshold of 5; (4) presence of diverse tumor types; and (5) occurrence of death or discharge within the initial 24 h post-ICU admission. After applying these exclusions, a total of 134 participants were deemed eligible for inclusion in the study (refer to Figure 1 for a detailed participant flow diagram).

### 2.2 Data acquisition

We meticulously extracted EMR data encompassing patient demographics, vital signs, and laboratory findings to delineate clinically distinct DIC phenotypes. To ensure that our clustering analysis was reflective of the data available at the time of ICU admission, we confined our data extraction to the initial 24-h window following ICU admission. In instances where multiple vital sign or laboratory measurements were recorded within this timeframe, we prioritized the initial recorded values. Laboratory results with missing data exceeding 30% were systematically excluded from our analysis. For any remaining gaps in data, we employed the K-Nearest Neighbors (KNN) imputation method to estimate missing values before their incorporation into the cluster analysis.

### 2.3 Statistical analysis

Data analysis in this study was conducted using STATA 16.0 and R software (version 4.4.2). Descriptive statistics, including mean and standard deviation (SD) for continuous variables and percentages for categorical variables, were employed to summarize the basic characteristics of the data. Continuous variables were compared using the Mann-Whitney test, while categorical variables were assessed using the Chi-square test or Fisher’s exact probability test, as appropriate. Variables with more than 20% missing data were excluded to mitigate potential bias. For variables with less than 20% missing data, missing values were imputed using the K-Nearest Neighbors (KNN) imputation method.

Correlation analyses were performed on parameters recorded within 24 h of ICU admission, utilizing the “ggcorrplot” package in R. Based on the correlation coefficients obtained, four variables were selected for unsupervised K-means clustering, which was conducted using the “ConsensusClusterPlus” package in R. The distinct subtypes of critically ill patients with DIC were identified through consensus K-means clustering, with the optimal number of clusters (K value) determined by employing the “elbow method,” “cumulative distribution function (CDF) plot,” and “consensus matrix.” High-dimensional data were reduced in dimensionality and visualized using three common techniques: Principal Component Analysis (PCA), t-Distributed Stochastic Neighbor Embedding (t-SNE), and Uniform Manifold Approximation and Projection (UMAP). Additionally, logistic regression analysis was employed to investigate the association between the identified subtypes and clinical outcomes. The results of the logistic regression analysis are presented as odds ratios (ORs) with their corresponding 95% confidence intervals (CIs).

## 3 Results

### 3.1 Patient screening and selection of clinical features

**FIGURE 2:**
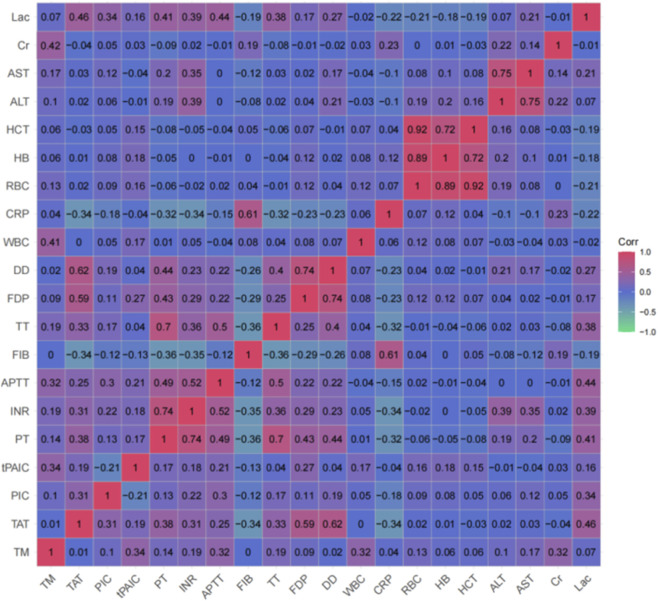
A heatmap of the correlation between candidate variables.

Out of the 3912 patients admitted to the ICU, a final cohort of 134 patients diagnosed with DIC was enrolled in the study (Figure 1). Initially, we generated heat maps using laboratory indicators collected within 24 h of ICU admission. Variables with a correlation coefficient greater than 0.4 were excluded to refine the dataset. Ultimately, four variables were retained for subsequent cluster analysis (Figure 2).

### 3.2 Identification of the optimal number of clusters

**FIGURE 3:**
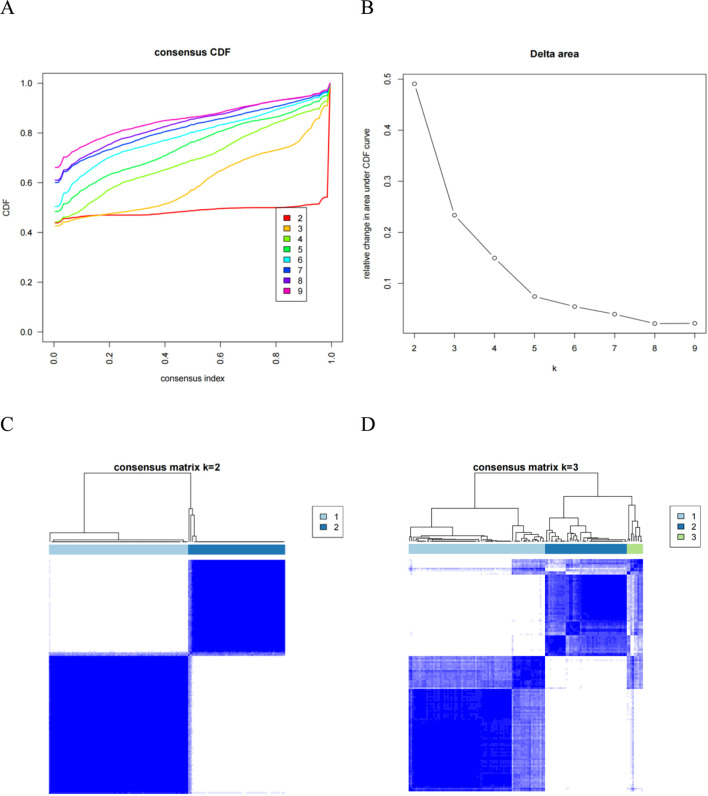
Analysis of the clustering process for critically ill patients with DIC through consensus clustering. **(A)** CDF plot; **(B)** Delta area plot; **(C,D)** CM heat map; consensus matrices for k = 2 and k = 3 show clearer separation at k = 2, indicating more stable clustering. Abbreviation: CDF: cumulative distribution function; CM: consensus matrix.

The Cumulative Distribution Function (CDF) plot illustrates the consensus distribution for each cluster (Figure 3A). The delta area plot, which represents the relative change under the CDF curve, shows the most significant changes occurring between k = 2 and k = 5 (Figure 3B). The consensus matrix (CM) heat map revealed that the machine learning (ML) algorithm successfully identified clusters 2 and 3, with distinct boundaries, indicating strong stability across multiple iterations (Figures 3C,D). In summary, the ML consensus clustering method applied to baseline characteristics at admission identified two clusters that most accurately represented the data.

**FIGURE 4:**
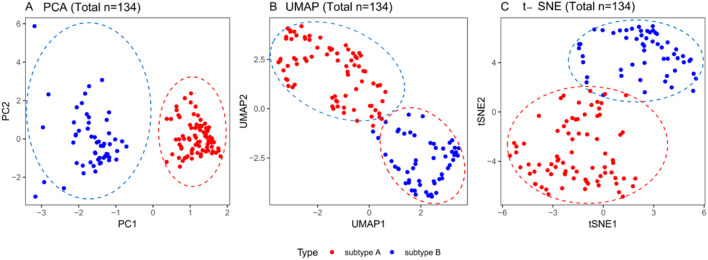
Using PCA, t-SNE, and UMAP methods to visualize DIC subtypes. **(A)** PCA; **(B)** UMAP; **(C)** t-SNE. Circles represent individual patients (blue represents Mild coagulation dysfunctional subtype (79 cases), red represents Severe coagulation dysfunctional subtype (55 cases)). Abbreviations: PCA, Principal Component Analysis; UMAP, Uniform Manifold Approximation and Projection; t-SNE,t-Distributed Stochastic Neighbor Embedding.

Two distinct clinical subtypes of critically ill patients with DIC were identified, designated as Subtype A and Subtype B. Principal Component Analysis (PCA), t-Distributed Stochastic Neighbor Embedding (t-SNE), and Uniform Manifold Approximation and Projection (UMAP) plots all demonstrated clear and separate clinical characteristics between these subtypes, highlighting significant differences in their clinical profiles (Figure 4).

### 3.3 Baseline characteristics and key features of participants between DIC subtypes

This study comprised 134 participants with a median age of 62 years, of which 62.7% were male. Consensus cluster analysis categorized the DIC patients into two distinct subtypes. Subtype A encompassed 79 patients (58.9%), while subtype B comprised 55 patients (41.1%). As delineated in Table 1, the two identified subtypes within the DIC cohort demonstrated significant disparities in their clinical characteristics. In comparison to subtype A, participants in subtype B exhibited elevated levels of temperature (T), heart rate (HR), C-reactive protein (CRP), prothrombin time (PT), Fibrin Degradation Products (FDP), international normalized ratio (INR), activated partial thrombin time (APTT), thrombin time (TT), D-dimer, alanine transaminase (ALT), aspartate transaminase (AST), Lactate (Lac), Acute Physiology and Chronic Health Evaluation II (APACHE II), and DIC, alongside reduced levels of Fibrinogen (FIB), Systolic Blood Pressure (SBP), and Diastolic Blood Pressure (DBP). No significant statistical differences were observed in terms of gender, age, white blood cells (WBC), Platelet (PLT), Hemoglobin (HB), and Sequential Organ Failure Assessment (SOFA).

**TABLE 1:** Baseline characteristics of the subtypes.

Item	ALL (n = 134)	Subtype A (n = 79)	Subtype B (n = 55)	P value	Q value
Male,n,%	84 (62.7)	48 (60.8)	36 (65.5)	0.580	0.660
Age, yr	62 (50, 76)	65 (52, 77)	58 (47, 72)	0.094	0.130
Sepsis	69 (52)	43 (54)	29 (53)	0.846	0.880
Trauma	31 (23)	17 (22)	14 (25)	0.595	0.700
Others	34 (25)	19 (24)	12 (22)	0.956	0.960
T,°C	36.5 (36.0, 37.3)	36.5 (35.5, 36.8)	36.7 (36.2, 37.3)	0.016	0.029
HR,beat/min	107 (89, 120)	101 (85, 118)	119 (102, 126)	0.001	0.003
SBP, mmHg	116 (92, 135)	120 (100, 140)	102 (88, 121)	0.002	0.005
DBP, mmHg	63 (53, 75)	65 (56, 78)	58 (40, 74)	0.018	0.031
TM, TU/mL	18.4 (11.5, 26.6)	20.3 (12.5, 28.9)	17.0 (8.7, 24.2)	0.185	0.230
TAT, ng/mL	47.1 (12.9, 120)	15.5 (8.5, 38.9)	120.0 (98.6, 120)	<0.001	<0.001
PIC, ug/mL	5.2 (1.4, 16.5)	3.2 (1.0, 8.8)	10.0 (2.9, 29.2)	<0.001	<0.001
tPAIC, ng/mL	13.4 (5.5, 31.5)	13.4 (4.0, 26.8)	14.0 (7.3, 44.4)	0.082	0.120
PT, s	22.7 (18.9, 33.6)	21.2 (18.7, 24.8)	29.8 (20.7, 80.0)	<0.001	0.002
INR	1.8 (1.5, 2.4)	1.7 (1.5, 2.0)	2.1 (1.6, 3.0)	0.004	0.009
APTT, s	46.7 (34.6, 73.1)	40.8 (33.9, 58.0)	65.3 (38.9, 92.2)	0.001	0.004
FIB, g/L	1.35 (0.81, 2.72)	1.78 (0.95, 3.22)	0.96 (0.64, 1.62)	<0.001	<0.001
TT, s	21.2 (17.6, 26.5)	19.0 (16.8, 23.4)	25.3 (19.8, 31.8)	<0.001	<0.001
FDP, mg/L	42.0 (17.9, 113.0)	25.1 (13.8, 51.5)	115.0 (60.1, 183.0)	<0.001	<0.001
D-dimer, μg/L	12.2 (6.1, 28.7)	7.01 (5.1, 13.7)	28.2 (14.4, 36.2)	<0.001	<0.001
WBC, × 10^12^/L	12.1 (7.2, 17.8)	11.7 (7.4, 18.0)	12.2 (7.10, 17.5)	0.862	0.860
CRP, mg/L	29.6 (6.1, 99.7)	72.1 (9.0, 149.0)	12.4 (2.7, 65.4)	<0.001	0.002
HGB, g/L	87 (68, 108)	87 (70, 110)	86 (67, 104)	0.797	0.800
PLT, × 10^9^/L	53 (28, 96.0)	48 (23, 92)	57 (33, 114)	0.175	0.22
ALT, U/L	90 (23, 393)	52 (17, 200)	193 (43, 591)	0.001	0.004
AST, U/L	157 (47, 885)	79.3 (38, 465)	472 (98, 1208)	<0.001	0.002
Cr, μmol/mL	148 (90, 247)	142 (845, 256)	149 (103, 212)	0.724	0.800
Lac, mmol/L	5.6 (2.4, 10.6)	3.0 (1.4, 7.4)	10.1 (5.2, 16.0)	<0.001	<0.001
SOFA	13 (9, 15)	12 (9, 15)	13 (11, 15)	0.100	0.140
APACHE II	27 (21, 31)	25 (20, 29)	29 (24, 34)	0.002	0.005
DIC	6 (5, 7)	6 (5, 6)	6 (5, 7)	0.004	0.002

Values are n (%), mean ± standard deviation or median (interquartile range), unless otherwise noted. Abbreviations: ALT, alanine transaminase; APACHE II, Acute Physiology and Chronic Health Evaluation II; APTT, activated partial thrombin time; AST, aspartate transaminase; CRP, C-reactive protein; INR, international normalized ratio; FIB, fibrinogen; FDP, fibrin degradation products; WBC, white blood cells; PT, prothrombin time; TT, thrombin time; Lac, Lactate; HB, hemoglobin; PLT, platelet; Cr, Creatinine; SBP, systolic blood pressure; DBP, diastolic blood pressure; HR, heart rate; TM, thrombomodulin; TAT, thrombin-Antithrombin Complex; PIC, Plasmin-α_2_-Plasmin Inhibitor Complex; tPAIC, tissue plasminogen activator-inhibitor complex; SOFA, sequential organ failure assessment.

**FIGURE 5:**
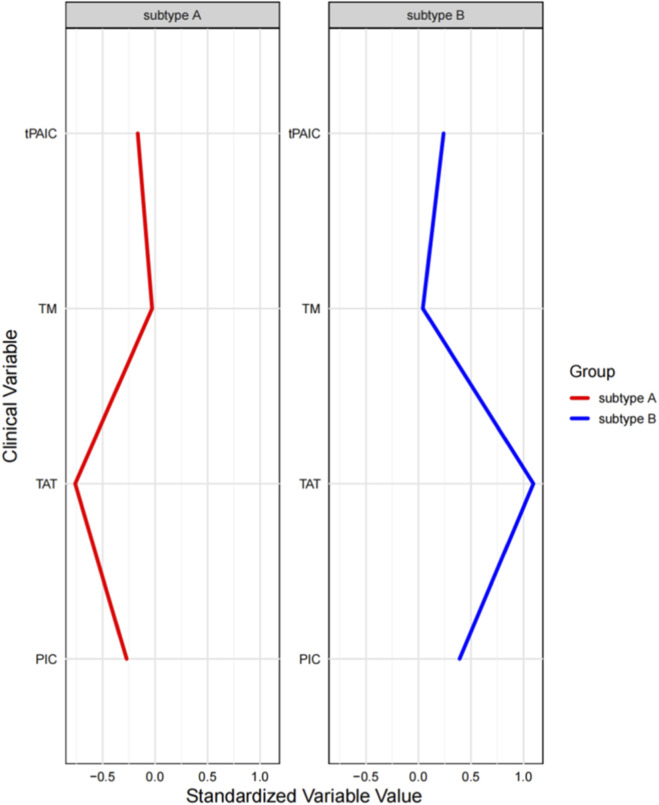
Baseline characteristics of four variables in DIC Subtype **(A,B)**. All variables are standardized to a mean of 0 and SD of 1. On the x-axis, a value of one denotes a phenotype mean 1 SD above the overall graph mean; −1 denotes 1 SD below. Abbreviations: SD, Standard Deviation.

The consensus clustering of baseline patient parameters revealed two distinct cohorts, whose multivariate profiles are depicted in the parallel coordinates plot in Figure 4. Key features distinguishing patients in subtype A from subtype B included lower thrombin-Antithrombin Complex (TAT), lower Plasmin-α_2_-Plasmin Inhibitor Complex (PIC), lower tissue plasminogen activator-inhibitor complex (tPAIC), and higher thrombomodulin (TM) (Figure 5). Based on their distinct clinical and laboratory profiles, we designated subtype A as the “Mild Coagulation Dysfunctional Subtype” and subtype B as the “Severe Coagulation Dysfunctional Subtype.”

### 3.4 Association of subtypes with outcomes

**FIGURE 6:**
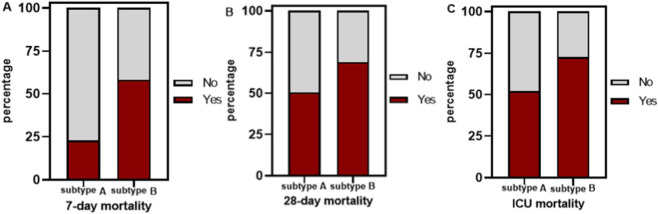
Comparative Analysis of Multiple Mortality Rates in DIC Subgroups A and B **(A)** 7-day mortality; **(B)** 28-day mortality; **(C)** ICU mortality. Abbreviation: ICU: intensive care unit.

During hospitalization, a subset of participants succumbed to their illness. The 7-day mortality, 28-day mortality, and ICU mortality rates for subtype A patients were 23%, 48%, and 52%, respectively, in contrast to 58%, 69%, and 73% for subtype B patients (all P < 0.05) (Figure 6).

**FIGURE 7:**
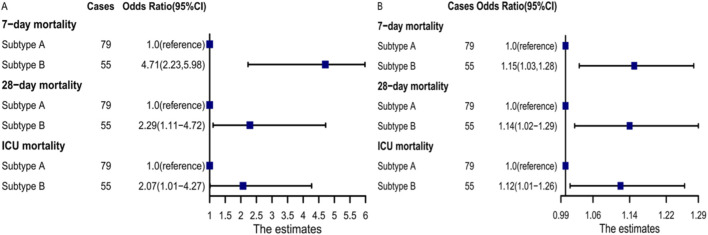
Logistic regression analysis for the association between subtypes and short-term outcomes. **(A)** Univariate logistic regression analysis. **(B)** Multivariable Logistic regression analysis, adjusting for age, gender, APTT, FIB, TT, FDP, DD, WBC, CRP, HB, PLT, ALT, AST, Cr, APACHEII, Lac, SOFA.

**FIGURE 8:**
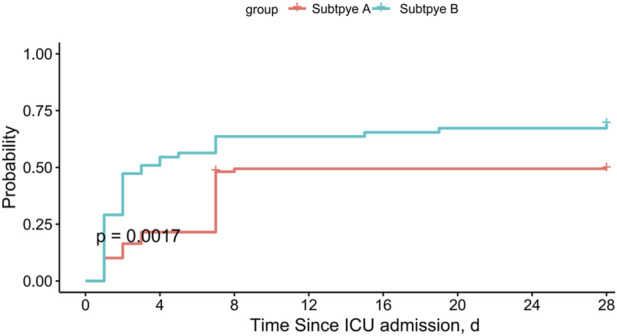
28-day mortality among different clusters of patients with DIC.

Logistic regression analysis revealed that subtype B was significantly associated with increased 7-day mortality (odds ratio [OR]: 4.71; 95% confidence interval [CI]: 2.23–5.98; *P < 0.001*), 28-day mortality (OR: 2.29; 95% CI: 1.11–4.72; *P= 0.024*), and ICU mortality (OR: 2.07; 95% CI: 1.01–4.27; *P= 0.047*) (Figure 7A). After adjusting for other covariates, subtype B was significantly associated with increased 7-day mortality (odds ratio [OR]: 1.15; 95% confidence interval [CI]: 1.03–1.28; *P= 0.13*), 28-day mortality (OR: 1.14; 95% CI: 1.02–1.29; *P= 0.020*), and ICU mortality (OR: 1.12; 95% CI: 1.01–1.26; *P= 0.038*) (Figure 7B). Subtype B was significantly associated with higher 28-day mortality when compared to subtype A (Figure 8).

## 4 Discussion

The deployment of unsupervised machine learning clustering methodologies significantly streamlines the analytical process of identifying and classifying patient phenotypes at the point of admission, utilizing targeted datasets (Serra-Burriel and Ames, 2022; Wang et al., 2023). In our study, the consensus clustering algorithm revealed two distinct subtypes within a cohort of critically ill patients with DIC. The differentiation of DIC phenotypes was anchored in the evaluation of TAT, PIC, TM and t-PAIC. Furthermore, These subtypes demonstrate statistically significant associations with multiple study endpoints, including 7-day, 28-day, and ICU mortality rates, indicating important correlations rather than direct causal relationships. This classification technique could offer valuable insights into the pathophysiology of DIC and may be considered for adoption as a risk assessment tool in clinical settings.

Previous scholarly work has explored the stratification of DIC (Wada and Gando, 2024; Wada et al., 2014; Taylor et al., 2001). Notably, the ISTH classification system classifies patients into two phenotypes based on coagulation assays, which are indicative of disease severity and associated outcomes (Taylor et al., 2001). The conventional “non-overt DIC and overt DIC” classification is the most frequently employed for DIC. This system assigns patients to one of two risk categories based on a scoring system with four variables, capped at eight points. Patients with scores below five points (non-overt DIC) are associated with a lower mortality risk, while those scoring five points or above are at a higher risk of death. Although this classification is designed for expedited diagnosis, the arbitrary cutoff values may introduce bias, potentially compromising the diagnosis and clinical management of DIC. As a result, there has been a continuous evolution in the proposal of new diagnostic criteria and classification methods for DIC. A recent classification distinguishes between thrombotic and fibrinolytic phenotypes, which present a discriminatory potential for predicting morbidity and mortality (Wada and Gando, 2024). These phenotypes are based on the extent of thrombosis and hemorrhage, facilitating the ongoing assessment of these risks throughout a patient’s hospital stay. However, it is important to recognize that these classification tools are primarily based on expert consensus and theoretical considerations rather than robust clinical evidence. Despite the existence of various classification systems, the mortality rate for DIC remains unacceptably high, underscoring the need for further research and refinement in this area.

In this study, we harnessed consensus clustering analysis to accurately classify DIC by revealing the underlying structure within multivariate data. This analytical technique is a mainstay in medical research, especially for characterizing clinical phenotypes, due to its ability to manage multiple variables autonomously while preserving the continuity of data. For instance, Cai et al. utilized clustering analysis to identify three distinct sepsis-induced coagulopathy (SIC) phenotypes influenced by heparin therapy (Cai et al., 2023). Our research, however, deviates from theirs in terms of the study population, statistical methodologies applied, and the consideration of multiple endpoints. Within our cohort, two distinct DIC phenotypes were delineated. Patients in subtype B, as opposed to those in subtype A, presented with deteriorated coagulation parameters, heightened scores on clinical assessment scales, and inferior clinical outcomes, indicating a greater propensity for multi-organ failure. The key variables differentiating DIC phenotypes included TAT, PIC, TM, and tPAIC, which have been implicated in mortality risk across various studies and are crucial for diagnosing DIC (Mei et al., 2019; Zhang et al., 2021). The significantly elevated TAT in subtype B signifies coagulation activation and the risk of thrombotic organ failure, whereas increased PIC and tPAIC indicate secondary hyperfibrinolysis and bleeding tendencies. This pathophysiological mechanism plunges patients into a state of “hypercoagulability with secondary hyperfibrinolysis,” simultaneously exhibiting both thrombotic and fibrinolytic phenotypes. This condition predisposes patients to a vicious cycle of diffuse microthrombosis and coagulation factor exhaustion, leading to progressive organ failure and uncontrollable hemorrhage, ultimately resulting in death. Our findings indicated that patients with subtype B faced significantly worse clinical outcomes than those with subtype A, exhibiting substantially elevated 7-day, 28-day, and ICU mortality rates. The association between DIC subtypes and the risk of short-term mortality was further validated by logistic regression analysis. Thus, classification through clustering enhances the predictive power of mortality and may guide clinical management for critically ill patients with DIC. In clinical practice, rapid detection of the aforementioned biomarker panel enables prompt phenotyping of DIC patients. Patients identified as Subtype B should be considered extremely high-risk, warranting immediate and intensified monitoring and intervention at the ICU bedside, including early enhanced heparinization therapy, organ function support, and dynamic assessment of treatment response to potentially improve clinical outcomes, rather than a conservative approach centered solely on replenishing coagulation factors.

The strengths of our study are highlighted by the innovative insights obtained through an unsupervised ML clustering approach. Guided by the investigation of pathophysiological mechanisms, this study identified subtypes of disseminated intravascular coagulation that can expedite patient assessment in the ICU, employing key features derived from rapid, simple, and cost-effective laboratory tests. However, our study is not without its limitations. Firstly, as a retrospective analysis, it is subject to the biases inherent in historical data. Secondly, the limited sample size and single-center design constrain the generalizability of this study’s findings, while the application of machine learning methods to small datasets increases overfitting risks and may compromise model validity, necessitating future validation through larger-scale prospective studies. Thirdly, the DIC classification subtypes identified in this study were not directly compared with the established thrombotic/fibrinolytic phenotypes in terms of their characteristics and clinical outcomes. Fourth, this conclusion may not be applicable to cancer patients. Fifth, as an observational study, this research did not fully control for the potential impact of treatment heterogeneity (such as differences in anticoagulation, transfusion, and organ support protocols) on the outcomes. Future research should focus on subgroup analyses based on the diverse etiologies of DIC.

## 5 Conclusion

In conclusion, our study demonstrates the utility of the consensus clustering algorithm in delineating distinct subgroups among hospitalized patients afflicted with DIC. This discovery highlights the potential of unsupervised machine learning methodologies in the domain of adult critical care. The DIC phenotypes characterized in our study were correlated with differential mortality rates at the 7-day, 28-day, and ICU admission milestones. These findings may serve as a valuable asset in the refinement of risk stratification and the formulation of targeted therapeutic strategies for DIC.

## Data Availability

The data analyzed in this study is subject to the following licenses/restrictions: The datasets generated during and/or analyzed during the current study are available from the corresponding author (Jingchun Song) on reasonable request. Requests to access these datasets should be directed to songjingchun@126.com.
